# Self-Medication Practices Among the Geriatric Population: A Systematic Literature Review

**DOI:** 10.7759/cureus.42282

**Published:** 2023-07-21

**Authors:** Khushal P Ghodkhande, Sonali G Choudhari, Abhay Gaidhane

**Affiliations:** 1 Epidemiology and Public Health, Jawaharlal Nehru Medical College, Datta Meghe Institute of Higher Education and Research, Wardha, IND; 2 Epidemiology & Public Health, Community Medicine, Jawaharlal Nehru Medical College, Datta Meghe Institute of Higher Education and Research, Wardha, IND; 3 School of Epidemiology and Public Health, Jawaharlal Nehru Medical College; Datta Meghe Institute of HIgher Education and Research, Wardha, IND

**Keywords:** drugs, non-prescription drugs, self-prescribed drugs, over the counter drugs, adverse drug reactions

## Abstract

The ageing population is increasingly using self-medication due to comorbidities. Most people who self-medicate use over-the-counter (OTC) medications bought from private pharmacies as their primary source of medicine. The use of self-medication may lead to an increased risk of unfavourable health outcomes. People over the age of 65 are more vulnerable to adverse drug reactions (ADRs). Our article aims to gain insights into self-medication in the geriatric population. We searched the Medical Literature Analysis and Retrieval System Online (MEDLINE) via Google Scholar and PubMed databases. The PubMed search technique was customised for each database and was as follows: (self-medication (Title/Abstract)) AND (geriatric (Title/Abstract) OR elderly (Title/Abstract) OR old (Title/Abstract)). Also, we used other databases like the World Health Organization (WHO), the Ministry of Health and Family Welfare(MOHFW) under the Government of India, etc. The keywords used for the search strategy were ‘over-the-counter drugs’, adverse drug reactions’, self-prescribed drugs’, and non-prescription drugs’. Articles that were not relevant to the review topic are excluded. Through our review, we found that most geriatric people use self-medication because of their previous experience with that medication, a lack of seriousness regarding the consequences of using OTC medications, and suggestions from family members, friends, or neighbours. Abdominal pain, headache, cough, joint pain, and fever are the conditions for which the geriatric age group mainly uses self-medication. The primary source of self-medication is directly from the pharmacy, and the most commonly consumed drug for self-medication is analgesics. Most people know about the risks associated with self-medication. However, people continue to participate in this risky self-medication behaviour to get quick relief from a mild illness. This issue can be resolved by providing such a group with free consultations or medical insurance. Pharmacists’ role in self-medication is also important. Counselling regarding the hazards of self-medication and selling the drugs to consumers without a doctor’s prescription must be avoided.

## Introduction and background

The World Health Organization (WHO) states that self-medication involves choosing and utilising medications for one's self-diagnosed ailments or illnesses [1]. Self-medication is the adoption and utilisation of medicine by people (or associates of people) to treat their self-reported or independently confirmed symptoms [2]. People use medications based solely on their knowledge and expertise instead of seeking a physician’s advice or a prescription. In fact, it also involves the use of family members’ medications, particularly when considering the care of the elderly [2,3]. Self-medication also entails using unused or possibly outdated and expired pharmaceuticals at home to treat a condition that one has self-diagnosed based on its symptoms [4].

According to the U.S. Food and Drug Administration (FDA), over-the-counter medications are effective medications that the general population can take without consulting a doctor, and it is safe to do so [5]. According to estimates, the number of over-the-counter (OTC) medications in the pharmaceutical market was more than 100,000 [6,7]. Private pharmacies are the main source of OTC medicines for most people in Riyadh, Saudi Arabia [8]. The management of minor illnesses is greatly aided by self-medication, which is prominent in the healthcare system. Professional medical advice is either prohibitively expensive or not easily accessible to the general public [9]. Without a prescription, using over-the-counter drugs is considered rational self-medication and an exercise that is acceptable around the globe [10]. However, taking medications without a prescription is risky, as they may develop severe and adverse effects [11]. Hence, the habit of self-medication might be dangerous. Most of those who self-medicated used allopathic medications, followed by ayurveda and homoeopathy [12].

People aged 60 and older are considered to be elderly [13]. The senior population is growing worldwide, even in developing nations. By 2025, there will be close to 1.2 billion older adults worldwide, up from 550 million in 1996 [13]. In most nations, an older society is expanding quicker than the overall population due to a birth rate reduction and a life expectancy increase. Ageing occurs more quickly in less developed countries than in more developed ones. In India, the number of people aged 60 and older is predicted to double between 2001 and 2026 [13]. Through this article, we focused on self-medication, particularly in the geriatric population, along with the adverse effects due to the consumption of medications like analgesics and antibiotics. Due to comorbidities, the ageing population is more likely to self-medicate, and they are unaware of the risks of frequently used self-medication [13]. They mostly use previously prescribed and over-the-counter drugs, increasing the risk of unfavourable health outcomes [14]. Due to the physiological changes in metabolism brought on by age, the older population is more vulnerable to adverse drug reactions (ADRs) [15]. A vital issue is antibiotic-related adverse effects [16]. The older population has a relatively high frequency of adverse drug reactions [6]. This review aims to gain insight into the reasons behind the use of self-medication by older people. The main objective is to determine the reasons for elderly individuals’ self-medication, followed by identifying the knowledge source of medications, awareness regarding potential health risks associated with self-medication overdoses, and the pharmaceuticals older people use most frequently for self-medication.

## Review

Methodology

This review focuses on awareness of self-medication practices among the geriatric population. For this article, to identify pertinent original and reviewed publications, we searched the Medical Literature Analysis and Retrieval System Online (MEDLINE) via Google Scholar and PubMed databases. The PubMed search technique was customised for each database and was as follows: (self-medication (Title/Abstract)) AND (geriatric (Title/Abstract) OR elderly (Title/Abstract) OR old (Title/Abstract)). The period filters were from 1998 to 2022. Also, we used other databases like those of the World Health Organisation (WHO), the Ministry of Health and Family Welfare (MOHFW) under the Government of India, etc. To find all relevant articles, several keywords and medical subject heading (MeSH) terms were used interchangeably and in combination, which included awareness of self-medication practice among elders. The key search terms used were ‘self-medication’, ‘awareness of self-medication’, ‘self-medication in elders’, 'over-the-counter drugs', 'non-prescription drugs', 'self-prescribed drugs', adverse drug reactions, etc. Original Article in English that assessed the cause of self-medication and checked its awareness among geriatric people.

**Figure 1:**
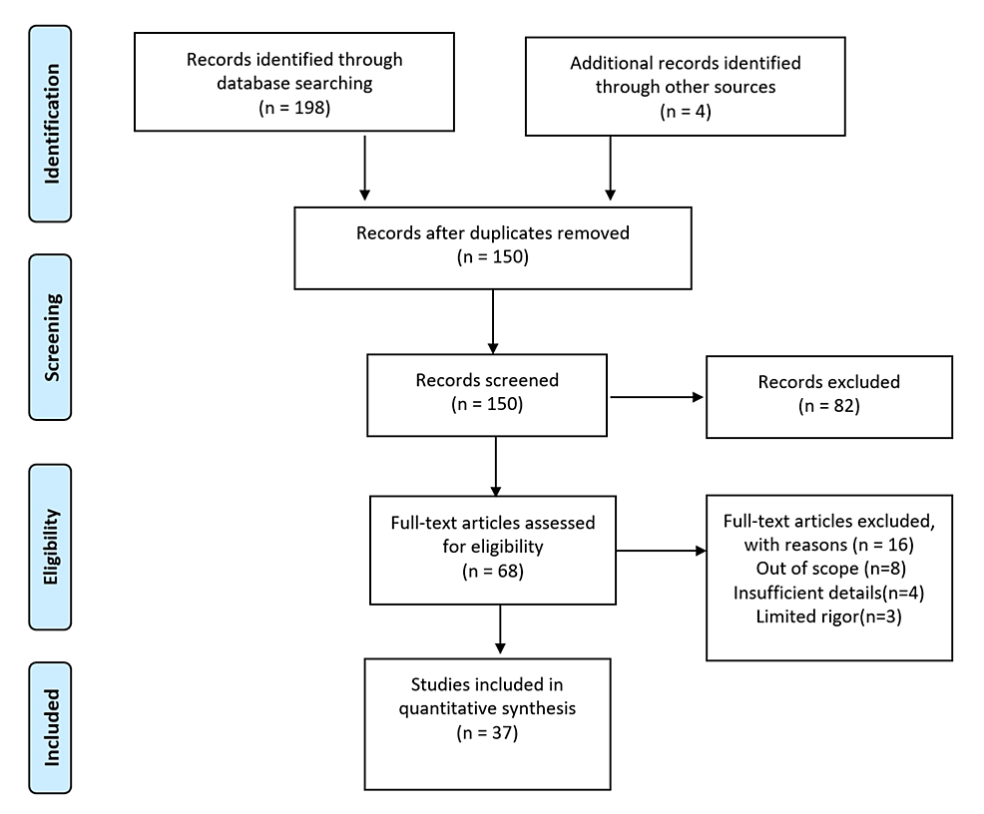
The study inclusion and exclusion criteria for this article

Articles of any kind were considered if they were deemed related to the subject of our review. The excluded articles were the ones that did not mention self-medication and the ones that were not available in the English language. One hundred ninety-eight records were obtained from the database, while four records were obtained from various other sources. After removing duplicate records, there were 150 records in total. Sixty-eight articles were left after being sorted by title, abstract, and full-text availability. According to inclusion and exclusion criteria, 37 papers were eventually included in this review’s synthesis of the evidence. Self-medication, its impact on elderly patients, and the adverse effects of OTC drug overdose have been the main topics of investigation. Figure 1 depicts the study inclusion and exclusion criteria for this article.

Self-medication trends around the world

Self-medication and excessive pharmaceutical use are common in various nations, including Iran, according to studies. The overall incidence of self-medication is 43.2%, 73.3%, and 59% in Ethiopia, Nigeria, and Nepal, respectively. On the other side, Iran is thought to have a three-times higher prevalence of self-medication than the rest of the globe [15]. About 13% of older patients in the US are hospitalised due to medication-related issues or pharmaceutical poisoning, which has killed 106,000 people and cost the American healthcare system USD 58 billion [17]. For upper respiratory tract infections (URTIs), about 24.2% of Chinese immigrants used antibiotics without consulting a doctor. If their symptoms subsided, over 70% of Chinese immigrants stated they would stop taking their medications; 61% of people would use any leftover antibiotics if they experienced the same symptoms. Furthermore, if individuals finished an incomplete course of antibiotics or used leftover drugs, consumers had a higher propensity to declare using antibiotics for self-medication. Members who believed in using antibiotics more frequently took self-medication for URTIs, fever, cough, and sore throat [18]. The study conducted in the Pokhara Valley of Nepal found that 38.2% of the clients used self-medication for the most frequent symptoms like headaches, and half of the people reported having physical aches. Colds and coughs were next (31%), followed by gastritis (23%) [19]. In Brazil, the rate of self-medication was 16.1%, the highest in the northeast region [20].

Use of self-medication in India

As the frequency of self-medication is high worldwide and also high in India. As per the study conducted in a Puducherry, India, urban area, self-medication was found to be 11.9% [21]. According to a study conducted in the urban area of Kerala, the rate of antibiotics used for self-medication was only 3.31% [22]. This rate is low compared to the studies done in India. This low rate of antibiotic use as self-medication in Kerala is because of the high literacy rate, which results in greater awareness of risk or side effects among the people of Kerala. A cross-sectional study in Kerala's urban population showed that males are more susceptible to self-medication than females. A similar trend was observed in the research carried out in Uttar Pradesh's remote regions [22,23]. In the Puducherry research, women reported using self-medication more frequently than men [21]. Research conducted in Delhi, India, found that 92.8% of people reported using self-medication, out of which 74.9% preferred allopathic medicines. It is found that young adults are more consumers of self-medication than older adults [24].

Why do people self-medicate?

Due to cognitive and physiological changes associated with increasing comorbidities, drug usage tends to increase [25]. Self-medication is quite prevalent, and there are many possible causes. The requirement for self-care is explained by the increasing trend of self-medication, a sympathetic attitude towards ailing family members, time constraints, financial limitations, lack of access to healthcare, illiteracy, and misinformation, as well as the prevalence of drugs outside of traditional drug stores and extensive advertising [26]. The cause might be anything from a lack of physicians to economic considerations [27].

Easily Affordable 

Since they cannot afford to see a doctor, many participants prefer to go to a local drugstore; some may not have insurance due to the cost. This makes individuals more likely to self-medicate [28].

Convenience 

Buying drugs from a local pharmacy is a more convenient option as compared to consulting a doctor and getting a prescription; most areas have access to community pharmacies within a short driving distance, and getting the required drugs takes little waiting time [28].

Perception of the Condition's Simplicity 

**Figure 2:**
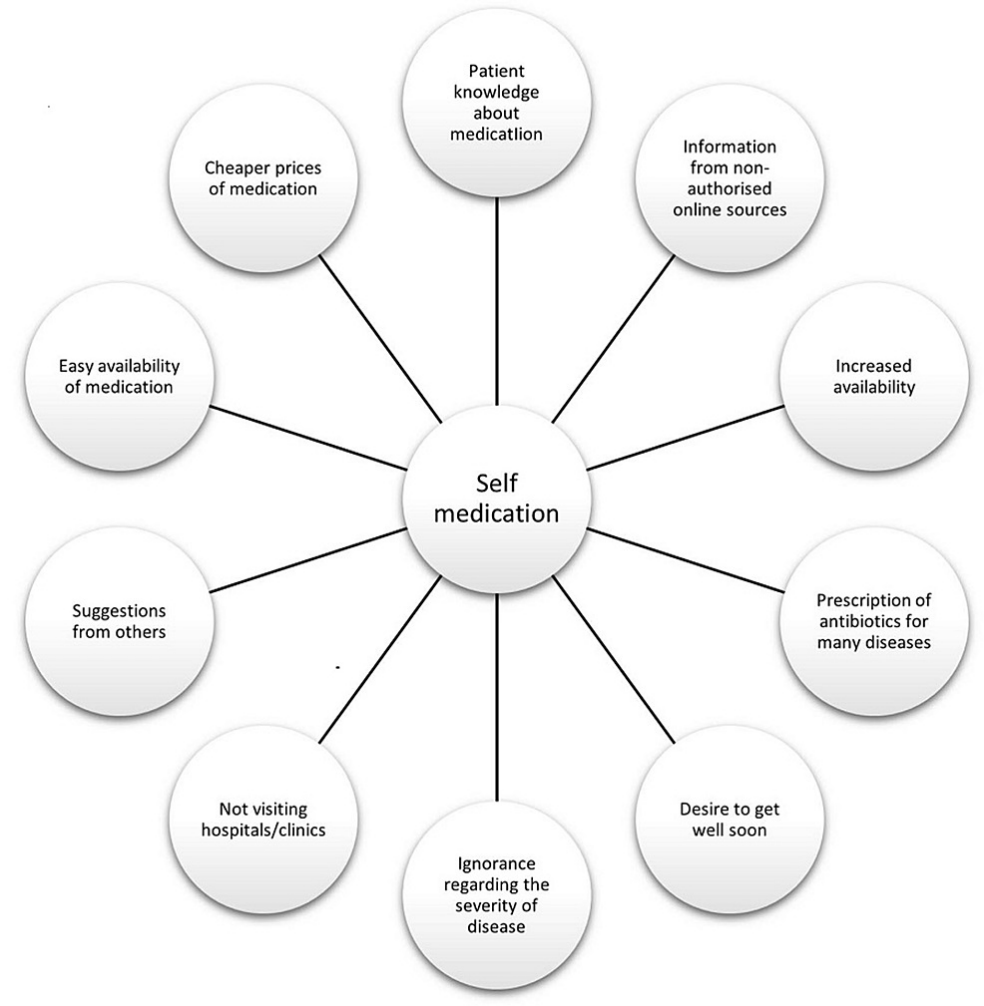
The participants' self-medication motives Adapted from [3]

Numerous people disclosed self-medicating for diseases they did not consider dangerous enough to need medical care. Illnesses are widespread and straightforward to identify [28]. Figure 2 depicts the participants' self-medicating motives [2].

Previous studies on the prevalence and pattern of self-medication in elders found that the most common use of self-medication is for abdominal pain, followed by headaches [13]. Behind taking self-medication in elders are other reasons, like repeating, common, or already experienced symptoms.

Sources of self-medication

Research done in a remote area of India found a percentage of sources from which people get information about medication for use without a prescription. Pharmacists (72.6%) were the primary source of self-medication information, followed by outdated prescriptions for prior illnesses (25.7%) and prescriptions from friends and family (1.8%) [27,29]. Table 1 depicts the common reasons, symptoms, sources, and drug groups used for self-medication [9,11,12,20,23-25,30,31].

**Table 1:** Common reasons, symptoms, sources of drug information, and drug groups used for self-medication by geriatric people. [9,11,12,20,23–25,30,31]

Common reasons for self-medicating	Common symptoms for which participants self-medicate	Common sources of drug information for self-medication	Common drug groups for self-medication
Minor Illness	Headache and other pain	Old prescription of the same illness	Analgesics
Avoidance of long waiting at clinics	Fever	Friends and family	Antipyretic
Convenience	Respiratory problems	Pharmacists	Antidiarrheal
Quicker relief	Cold and cough	Advertisement	Antibiotics
Economical (cheaper)	Acid reflux	Own experiences	Antacids
Urgency	Vomiting	Reading material	Antitussives
Time-saving	Diarrhoea	Social media	Antacids

Potential risks

The practice of self-medication is associated with several dangers for patients and the general public [30]. The hazards of self-medication are numerous. In particular, the average user typically lacks specialised knowledge of pharmacology, therapy, or the medication’s unique properties. The majority of people who use antibiotics for themselves lack the necessary dosage information and use them for an inappropriate amount of time, according to WHO reports. This practice may lead to the development of drug resistance [32,33].

This results in certain potential risks for the individual consumer; false inaccurate diagnosis, delaying seeking early, sound medical advice, inappropriate therapeutic approach, not understanding specific pharmacological hazards, rare but harmful side effects, drug-drug interactions, cautions, warnings and contraindications, or self-diagnosing them, ignoring the same active component under a different name currently being consumed, failure to inform the prescribing doctor about current self-medication, not identifying or reporting adverse medication reactions, incorrect administration route or method, excessive or inadequate dosage, decreased renal function, excessively extended use, abuse and dependence danger, risks in work, drug-food interactions, improper storage methods or storing and using items past their recommended expiration dates. Inappropriate self-medication could increase drug-induced disease and unnecessary public spending at the community level [34].

Managing the health hazards of self-medication; the function of medical experts

Information

Medical experts must always provide patients with clear instructions and explanations when prescribing drugs to help them comprehend and enable them to make their own decisions. Details ought to be given to patients at their level of understanding and assessed for health literacy skills and the use of pictograms or pictures for low literacy patients so they can understand how to handle it because older adults may have difficulty reading the small print on medication labels [26].

Education

Health officials should prioritise the public's health education activities [34]. Patients' incorrect and irregular self-medication and non-adherence may only be decreased if they are educated and comprehend the logic behind specific advice. The scope of non-compliance with inaccurate and inconsistent self-medication by clients may only be decreased if they are informed and educated about the logic behind certain advice [26].

Therapeutic Advice

Lack of therapeutic adherence, which arises from an inadequate or insufficient description of the treatment goals, is a severe issue in acute and chronic treatments. Patients are less likely to use the medication correctly if they are not informed. However, if the usage instructions and restrictions for a specific medication are explained, such as the dose, frequency of doses, length of treatment, ways to use it, etc., then clients follow universal principles to ensure proper pharmaceutical use both now and in the future [35].

The role of the pharmacist

Self-medication practices are widespread in society for several reasons. It is clear that improper self-medication habits exist and could harm patient care outcomes [36]. Unreasonable drug usage is becoming a more significant threat to public health [28]. Pharmacists are essential in improving patient education on self-care and medication use [28]. They play a crucial role in training their clients to take medications meant for self-medication. To accomplish this, they must undergo the necessary instruction and practice [26]. Drug regulatory organisations should ensure that all pharmacies and pharmacists are registered, that only doctors' prescriptions are needed to get restricted substances, and that rules protecting drug usage are appropriately implemented [34,37]. Pharmacists should encourage their clients to consult a physician before using any medicine for self-medication [8].

## Conclusions

In today's scenario, most people know about the risks associated with self-medication. However, people continue to participate in this risky self-medication behaviour to get quick relief from a mild illness. The older population often and heavily uses self-medication. This is concerning since seniors are more likely to experience negative medication responses than younger people. The predominant factor behind the use of self-medication is prior experience with that drug, and the most common symptom that is treated is a headache. Self-medication is most frequently obtained from pharmacies by telling symptoms to the pharmacists, and the economy and financial issues are the main contributors. However, this issue can be resolved by providing such a group with free consultations or medical insurance. The role of medical specialists in resolving self-medication is to provide information, therapeutic advice, and education. Pharmacists' role in self-medication is also important, by avoiding selling and giving training to the client who self-medicates. Unreasonable drug use is becoming a more significant threat to public health. Policymakers, programme administrators, and researchers would benefit from the study's findings by having a clearer understanding of the issues surrounding self-medication.
